# Antioxidant Activity of Essential Oils

**DOI:** 10.3390/antiox12020383

**Published:** 2023-02-05

**Authors:** Delia Mirela Tit, Simona Gabriela Bungau

**Affiliations:** 1 Department of Pharmacy, Faculty of Medicine and Pharmacy, University of Oradea, 410028 Oradea, Romania; 2 Doctoral School of Biomedical Sciences, University of Oradea, 410087 Oradea, Romania

In recent few years, the high efficacy of herbal antioxidant products in various diseases has been reported. Numerous essential oils (EOs) with antioxidant properties must be mentioned here, their use as natural antioxidants being a field of real interest—especially in food science and medicine. EOs are complex products, usually consisting of over 50 components in varying concentrations. The EO substances described as having antioxidant activity represent an important fraction of the total compounds, often being indicated as a potential source in the discovery and development of new bioactive compounds, with medical, pharmaceutical, cosmetic, and other uses. Additionally, EOs and their constituents have been studied as alternative additives in the food industry, emphasizing their advantages over synthetic antioxidants that often have negative effects on health.

This Special Issue collected original research articles and reviews dealing with all aspects of the antioxidant activity of EOs:

- The chemistry and mechanisms at the basis of EOs’ antioxidant activity;
- Methods used to measure antioxidant activity;
- In vitro and in vivo antioxidant activity;
- The pharmaceutical, cosmetic, and food applications of bioactive compounds from EOs and their mechanisms, focusing on their antioxidant activity;
- The testing of combinations of oils and combinations with other antioxidant compounds in order to increase their antioxidant potential;
- The antioxidant activity of innovative formulations, such as active packaging and nano/microparticles containing EOs.

A summary of the present information on this topic is provided by the 15 original research articles and two reviews gathered in this Special Issue.

In one review, Sharma et al. show that *Embelia ribes* may prove to be an efficient medicinal tool in the treatment of many illnesses, due to the abundance and variety of bioactive compounds that demonstrate antioxidant, wound-healing, anti-diabetic, antiviral, antibacterial, antifungal, anti-obesity, cardioprotective, and antifertility action, among other interesting pharmacological properties, such as that related to diseases of the central nervous system [1].

Another review, published by Agarwal et al., provides a thorough overview of the characteristics, content, and mechanism of action of citrus EOs in relation to a variety of health-related conditions. The soothing, calming, mood-lifting, and cheer-enhancing benefits of citrus EOs are coupled with their pleasant smell. Their dispersion generates a feeling of freshness and cleanliness, helps relieve tension and anxiety, and boosts mood and physical and emotional energy [2].

The effects of aromatherapy with EOs were also tested by Mot et al., in a study determining the chemotype, antioxidant activity, and prospective aromatherapy uses for *Salvia officinalis* (common sage) EO, in a medical setting. According to this investigation, EO has a limited antioxidant capability, but hospitalized patients may find that inhaling the oil’s high borneol content increases their comfort/pleasure [3].

In silico methods have been used by several authors, alongside classic in vitro methods, to test the antioxidant action and safety of the EOs/bioactive compounds studied. The study conducted by Karakoti et al. revealed some intriguing biological properties of three Vitex species (*V. agnus-castus*, *V. negundo*, and *V. trifolia*), particularly as natural phytotoxic agents and antioxidants, which supports the utilization of this plant species in traditional medicine beyond the domain of crop protection [4]. In the EO of *Allium sativum* cultivated in Peru, Herrera-Calderon et al. determined the presence of two oxygenated terpenes (α-bisabolol and an unknown constituent with the formula C_22_H_42_O_4_), not reported in other studies, associated by the authors with the high antioxidant power of the studied oil. Additionally, in silico studies (on nicotinamide adenine dinucleotide phosphate oxidase) showed that α-bisabolol had the highest docking rating and displayed excellent stability. According to the ADMET prediction, all garlic components can be administered orally and topically without causing any toxicity [5]. According to the results of the research carried out by Minchán-Herrera et al., volatile *Valeriana pilosa* components may contribute to the observed antioxidant effect by functioning as potential CYP2C9 gene and xanthine oxidase inhibitors [6]. Shah et al. reported, for the first time, the pharmacological activities of *Scutellaria edelbergii* EOs. The results indicate the presence of the bioactive constituent methyl 7-abieten-18-oate, which has the potential to function as an antioxidant, efficient painkiller, and anti-inflammatory agent, in addition to being a prospective candidate molecule for use against microorganisms. The anti-inflammatory ability of methyl 7-abieten-18-oate to inhibit cyclooxygenase-2 enzyme activity has been further validated by computational research, and its absorption, distribution, metabolism, excretion, and toxicity (ADMET) properties support the use of this molecule for additional investigation in a clinical trial [7].

Cytotoxic assays against *Artemia salina* were performed in three studies. New information is presented by Cascaes et al. regarding the antioxidant activity, chemical composition, and early toxicity of EOs from the Brazilian Amazonian species Duguetia and Xylopia (Annonaceae). When compared with EO derived from D. riparia, which had low toxicity or was non-toxic, EOs obtained from *D. echinophora, X. frutescens*, and *X. emarginata* showed significant toxicity. The EOs of *X. frutescens* and *X. emarginata* had the strongest ability to neutralize 2,2-diphenyl-1-picrylhydrazyl (DPPH) and 2,2′-azino-bis(3-ethylbenzothiazoline-6-sulfonic acid (ABTS) radicals. The primary components of these EOs may be predominantly responsible for the observed antioxidant capacity, but the influence of minor constituents should also be mentioned [8]. *Myrcia sylvatica* and *Myrciaria floribunda* (Myrtaceae) EOs were studied by de Moraes et al. According to preliminary toxicity studies, *M. floribunda* EO was moderately toxic compared with *A. salina*, whereas *M. sylvatica* EO was very toxic. Furthermore, *M. floribunda* EO demonstrated a stronger ability to block the DPPH radical [9]. The first study on the chemical content, antioxidant properties, and initial toxicity of the Amazonian plant extract known as *Croton campinarensis* is presented by da Costa et al. The EO aromatic profile was represented by terpenes, with a prevalence of sesquiterpene hydrocarbons (87.95%). A significant suppression of DPPH radicals was seen in the Trolox Equivalent Antioxidant Capacity assessment. According to the initial cytotoxicity assay against *A. salina*, the EO of *C. campinarensis* has a lethal concentration of 20.84 ± 4.84 µg·mL^−1^, making it toxic [10].

In vitro and in vivo studies were performed to test the biological effects of *Artemisia judaica L*. and *A. visnaga* L. EOs and *Heracleum persicum* oil nanoemulsion. Mohamed et al. carried out phytochemical investigation of *Artemisia judaica* L. for its antioxidant and anti-inflammatory properties, high concentration of oxygenated monoterpenes and cinnamate derivatives, and its therapeutic effect in the treatment of skin wounds [11]. *A. visnaga* L’s chemical content and antioxidant capacities were investigated by Kamal et al. Although in vitro studies indicated a low antioxidant potential, *A. visnaga* L. EO supplementation considerably enhanced antioxidant capacity, as evidenced by an increase in the antioxidant enzyme activities of catalase, superoxide dismutase, and plasma glutathione peroxidase, and a decrease in the levels of 3,4-methylenedioxyamphetamine (MDA), according to an in vivo investigation on Swiss albino mice [12]. Bashlouei et al. prepared *Heracleum persicum* oil nanoemulsion (HAE-NE) and investigated its biological properties against healthy human fibroblasts from the foreskin and human breast cancer cells. In the liver, kidney, and jejunum of mice, the HAE-NE at 1.5, 2.5, and 3.5 g/concentration increased caspase 3 and accelerated the sub-G1 peak of the cell cycle with no harmful effects. The results indicate that HAE-NE presented the ability to be turned into therapeutic medications and might represent an environmentally friendly nanotherapeutic alternative for use in food, cosmetics, and pharmaceutical applications [13].

Potential applications in the aquaculture sector were tested by Magara et al., by evaluating the regulation of antioxidant defense against oxidative stress in the rainbow trout, *Oncorhynchus mykiss*, fed fish meal supplemented with a supercritical extract of basil (F1-BEO). Increased F1-BEO supplementation in fish meal (1–3% *w/w*) led to decreased glutathione levels and the failure of numerous important antioxidant enzymes. Furthermore, the levels of MDA indicate that fish fed diets containing 0.5–2% (*w/w*) F1-BEO supplements had sufficient oxidative stress defense supported by antioxidant pathways over the experimental period. When trout are administered highly substituted meals with F1-BEO for prolonged periods of time, the decline in crucial stress-shielding molecules raises the alarm for possible oxidative damage [14].

To improve the quality attributes of Marchigiana Burgers, the oxidative stability, color characteristics, microbiological profile, and fatty acid content of burgers treated with and without a combination of EOs (*Origanum vulgare var. hirtum* and *Rosmarinus officinalis*) were all evaluated by Fusaro et al. in their study on young Marchigiana bulls. The use of EO had an impact on the amount of thiobarbituric acid reactive material (*p* < 0.05). EOS, however, had no impact on the microbial profile or color characteristics while storing meat [15]. Manzur et al. evaluated the antioxidant and antipathogenic properties of the commercial EOs from orange—*Citrus sinensis* (L.) Osbeck—and concluded that these oils may serve as natural and secure substitutes to increase the period of validity of foods by preventing contamination and oxidation with pathogens that ruin food. Additionally, sweet orange EOs may offer an inventive dual strategy for food conservation [16]. The rosemary extracts derived from raw materials, post-distillation materials, and post-supercritical CO_2_ extraction (scCO_2_) materials were all initially compared by Luca et al., using a phytochemical and multi-biological method. According to the authors, terpene-rich extracts (EO, scCO_2_) that are typically utilized as aroma-active substances or food preservatives (antioxidants) can be replaced with byproducts [17].

The articles collected here offer fresh perspectives on the expanding knowledge and research possibilities in the creation of new nutraceutical/adjunctive approaches, taking advantage of the antioxidant potential of the bioactive chemicals found in EOs.
